# Predictors of provider- initiated HIV testing and counseling refusal by outpatient department clients in Wolaita zone, Southern Ethiopia: a case control study

**DOI:** 10.1186/s12889-016-3452-8

**Published:** 2016-08-12

**Authors:** Wolde Facha, Wondewosen Kassahun, Abdulhalik Workicho

**Affiliations:** 1 School of Public Health, College of Health Sciences and Medicine, Wolaita Sodo University, Wolaita Sodo, P.O.Box 138, Ethiopia; 2 Department of Epidemiology, College of Health Sciences and Medicine, Jimma University, Jimma, P.O.Box 378, Ethiopia

**Keywords:** Provider-initiated HIV testing and counseling, PITC, HIV testing, Wolaita zone, Southern Ethiopia

## Abstract

**Background:**

Despite different strategies designed to rapidly identify HIV infected individuals, majority of HIV-infected people are unaware of their sero-status in developing countries. The objective of this study was to assess predictors of provider-initiated HIV testing and counseling (PITC) refusal by outpatient department (OPD) clients in Wolaita zone, Southern Ethiopia.

**Methods:**

Facility based unmatched case control study was conducted on outpatient department clients in randomly selected seven health facilities in Wolaita zone, Southern Ethiopia in February 2012. A total of 291 participants (97 cases and 194 controls) were included in our study. Cases were patients who refused HIV test while controls were patients who tested for HIV after provider-initiated HIV testing and counseling (PITC) recommendation by outpatient department (OPD) clinicians. We used both quantitative and qualitative methods of data collection. Pretested interviewer administered questionnaires were used to collect quantitative data by trained nurses, and in-depth interview with 14 OPD clinicians was conducted by principal investigator to supplement quantitative findings. Bivariate and multivariate analyses were done to identify independent predictors of provider-initiated HIV testing and counseling refusal by OPD clients.

**Result:**

Study participants who had stigmatizing attitude [AOR = 6.09, (95 % CI: 1.70, 21.76)], who had perceived risk for HIV infection [AOR = 5.23, (95 % CI: 2.22, 12.32)], who did not perceive the benefits of provider-initiated HIV testing and counseling [AOR = 4.64, (95 % CI: 1.79, 12.01)], who did not get minimum recommended pretest information from their providers [AOR = 2.98, (95 % CI: 1.06, 8.35)], who ever not heard of provider-initiated HIV testing and counseling service [AOR = 2.41, (95 % CI: 1.14, 5.09)], and who were from urban area [AOR = 2.40, (95 % CI = 1.26, 4.57)] were more likely to refuse provider-initiated HIV testing and counseling service than their counterparts.

**Conclusion:**

Knowledge on HIV/AIDS, attitude towards people living with HIV/AIDS and perceived risk for HIV infection by clients were the major barriers for provider-initiated HIV testing and counseling acceptance. Health professionals working at outpatient department should give due attention to overcome these barriers so as to enhance HIV testing acceptance by their clients.

## Background

About 34 million people (0.5 % of the world population) were living with HIV/AIDS in 2010 (estimates range from 30.9 to 36.9 million) [1]. Sub-Saharan Africa remains the most heavily affected region, accounting for 71 % of all new HIV infections in 2008 [2]. However, only 10 % of men and 12 % of women had been tested for HIV, implying that the majority of HIV- infected persons in this region are unaware of their sero status [2, 3]. With an estimated 1.1 million people living with HIV, Ethiopia is one of the 22 most affected countries in the world [4, 5]. The Government of Ethiopia set national HIV/AIDS policy in 1998 to create an enabling environment to fight the pandemic [6–8]. However, most HIV-infected patients globally, and particularly in resource-poor settings, are currently unaware of their HIV status due to various reasons [9]. Among all types of HIV testing, provider-initiated HIV testing and counseling (PITC) has been identified as a priority intervention to increase access to treatment, care and support for HIV infected individuals [9–11]. Provider-initiated HIV testing and counseling is HIV testing and counseling service recommended by health care provider to clients attending health facility as a standard component of medical care. Previously it was given at outpatient department (OPD) for patients with possible sign of HIV infection but now, as per WHO recommendation, the service is given to all clients as standard components of medical care not to miss HIV positive individuals seeking medical care [12–15]. After the introduction of the service, PITC refusal among adult patients at OPD was 5–25 % in different African countries [16, 17]. However, study conducted in our country even with possible sign of HIV infection showed that its refusal was about 32 % [18]. Thus, the government of Ethiopia has been using different strategies to encourage people to be tested for HIV. These includes escalating static and mobile voluntary counseling and testing (VCT) site, strengthening prevention of mother-to-child transmission (PMTCT) service, conducting campaign for HIV testing, enrolling HIV positive individuals to care and treatment program free of charge, etc. [10]. Despite these efforts to enhance HIV teting many people are still unaware of their sero-status which might be contributed by several factors. However, most of the researches conducted on PITC utilization addressed patients’ related factors. However, patients’ related factors were not the only factors affecting PITC refusal. Therefore, this study assessed both patients’ and providers’ related factors affecting PITC refusal at selected health facilities in Wolaita zone, Southern Ethiopia.

## Methods

### Study design and setting

We conducted facility based unmatched case–control study in February, 2012 in Wolaita zone, Southern Ethiopia. Wolaita zone is administratively divided in to twelve districts and three town administrations. Based on the 2007 census conducted by the Central Statistical Agency of Ethiopia (CSA), the population of Wolaita zone was projected to be 1,901,112 for the year 2012/13 (making it one of the most densely populated areas in the country). Among them, 96.31 % were Wolaita ethnic groups, 49.2 % were men and 11.49 % were urban inhabitants. (Urban was defined as localities of 2,000 or more inhabitants) [19]. Wolaita zone is located at a distance of 328 km south of the capital city (Addis Ababa). The zonal town (Soddo) is pathway for many travellers and tourists since it has five transportation gates to the nearby zonal towns. The major economic activities are agriculture (production of legumes, root crops and some cereals - predominantly maize), and livestock rearing which is source of income for about 88.5 % of population [20]. The zone has three hospitals, 71 health centers, 372 health posts and 98 private clinics. All hospitals and health centers were providing PITC service for OPD patients; however only three hospitals and thirteen health centers were providing antiretroviral therapy (ART) during the study period. We randomly selected two hospitals and five health centers among those facilities having HIV care and treatment programs.

### Study participants

Study population for cases were adult patients (age 15–64 years) who refused HIV test while for controls were adult patients who were tested for HIV after provider- initiated HIV testing and counseling recommendation by OPD clinicians. Cases were adult patients who refused HIV test while controls were adult patients who were tested for HIV after provider-initiated HIV testing and counseling recommendation by OPD clinicians.

### Sample size and sampling technique

We used both quantitative and qualitative methods of data collection. Sample size for quantitative study was calculated using OpenEpi (Epidemiologic Statistics for Public Health, version 2.2) by using proportion of cases and controls with high perceived stigma towards people living with HIV/AIDS (PLWHAs) of 69.15 and 38.29 % respectively [21], confidence interval (CI) of 95 %, power of 90 %, case to control ratio of 1:2 [18], design effect of 2 and 10 % possible non-response rate. Thus, the total of 291 OPD clients (97 cases and 194 controls) were participated in quantitative study and 14 OPD clinicians (two from each facility) were purposely included for in-depth interview. Study subjects were allocated proportionally to the respective health facilities based on patients flow and selected consecutively until the expected sample size was obtained.

### Data collection and measurement

We used Amharic and Wolaytigna (local language) version questionnaire to collect data from patients who had willingness to be included in the study at private room arranged for this purpose. Seven diploma nurses for data collection and four supervisors who have first degree in public health were used after 2 days training. Questionnaire was pre-tested on 10 % of clients at Sodo Christian Hospital which was not included in the study, and discussion was held based on the result of the pre-test and necessary correction was done. The questionnaire addressed socio demographic characteristics, knowledge on HIV, KAP on PITC, perceived benefits for HIV test, perceived risk to HIV infection and HIV related stigma to assess patients’ related factors; and whether the providers have given minimum recommended pretest information, post-test counseling, linked HIV positive patients to care, treatment and support or not were used to assess providers’ related factors. For qualitative study, in-depth interview with OPD clinicians were conducted to assess patients’ and providers’ related factors. It focused mainly on how PITC was implemented at their health facility, major facilitators and barriers to PITC service by patients, type of services delivered to HIV positive patients and problems faced while implementing PITC service at their health facility. Note taking and digital record was conducted during in-depth interview not to miss important points. Knowledge on HIV status was assessed by five questions related to HIV prevention and common misconceptions and those who answered all questions correctly were categorized as knowledgeable otherwise not knowledgeable. Stigma towards people living with HIV/AIDS was assessed using five questions. Those who were no stigmatizing idea for all five questions were categorized as no stigma while those individuals who have stigmatizing idea for at least one of five questions related to stigma were categorized as having stigma towards people living with HIV/AIDS. Those respondents who believed that PITC is beneficial for themselves were categorized as having perceived benefit for HIV testing while those respondents with feeling of vulnerability of being infected by HIV were operationalized as having perceived risk for HIV infection. Minimum recommended pretest informations on PITC are the informations on HIV transmission and prevention methods, patients’ right to decline HIV test, benefits and confidentiality of HIV test given to patients by OPD clinicians.

### Data analysis

Data was checked for completeness, edited, coded and entered into Epi data version 3.1 and exported to SPSS 16.0 statistical software for analysis. Double entry was done by principal investigator before analysis. Descriptive analysis was made to explain summary values and characteristics of participants. Bivariate analysis was done and all explanatory variables which have association with the outcome variable with p value less than 0.2 were included in multivariate analysis. Then multivariate analysis using stepwise selection method (backward LR) was employed to determine independent predictors among explanatory variables for PITC refusal. OR with 95 % CI were used to decide whether those independent variables included in multivariate analysis were statistically significant or not with outcome variable (PITC refusal). Finally qualitative findings were categorized on key thematic areas to supplement quantitative findings.

## Results

A total of 291 OPD clients (97 cases and 194 controls) were selected for our study and the response rate was 100 %.

### Socio-demographic characteristics

About 73 % of cases as well as controls were in the age group between 15 to 34 years, and the median age for cases was 25 years and that for controls was 27 years. About 53.6 % of cases and 51 % of controls were female in which 46.4 % of cases and 55.7 % of controls live in rural areas. Majority of study participants were married (51.5 % for cases and 67 % for controls), attended formal education (77.3 % for cases and 84.5 % for controls) and unemployed (78.4 % for cases and 81.4 % for controls) (Table 1).

**Table 1:** Socio-demographic characteristics of patients attending OPD of selected health facilities in Wolaita zone, Southern Ethiopia, February 2012

Variable	Case (*n* = 97) # (%)	Control (*n* = 194) # (%)	*P*-value
Age			0.509
15–24	41 (42.3)	70 (36.1)	
25–34	30 (30.9)	72 (37.1)	
35–64	26 (26.8)	52 (26.8)	
Sex			0.678
Male	45 (46.4)	95 (49)	
Female	52 (53.6)	99 (51)	
Residence			0.136
Rural	45 (46.4)	108 (55.7)	
Urban	52 (53.6)	86 (44.3)	
Marital status			0.031
Married	50 (51.5)	130 (67)	
Single	39 (40.2)	56 (28.9)	
Divorced/Widowed	8 (8.2)	8 (4.1)	
Formal education			0.132
No	22 (22.7)	30 (15.5)	
Yes	75 (77.3)	164 (84.5)	
Occupation			0.53
Employed	21 (21.6)	36 (18.6)	
Unemployed	76 (78.4)	158 (81.4)	
Av. household income			0.184
< 500	25 (25.8)	30 (15.5)	
501–1000	36 (37.1)	77 (39.7)	
1001–1500	25 (25.8)	56 (28.9)	
> 1501	11 (11.3)	31 (16.0)	

#### Knowledge on HIV prevention and common misconceptions

About 40.2 % of cases and 23.7 % of controls did not believe that the risk of HIV transmission is reduced by having sex with only one faithful uninfected partner. Regarding their knowledge on HIV prevention and major misconceptions, 74.2 % of cases and 62.4 % of controls were not knowledgeable (Table 2).

**Table 2:** Knowledge on HIV prevention and common misconceptions by cases and controls who attended OPD of selected health facilities in Wolaita zone, Southern Ethiopia, February 2012

Variables	Case (*n* = 97) # (%)	Control (*n* = 194) # (%)	Crude OR (95 % CI)
Faithfulness to partner reduce the risk of HIV transmission			
Yes	58 (59.8)	148 (76.3)	1
No	39 (40.2)	46 (23.7)	**2.16 (1.28,3.65)** ^a^
Consistent use of condom reduce the risk of HIV transmission			
Yes	50 (51.5)	125 (64.4)	1
No	47 (48.5)	69 (35.6)	**1.7 (1.04,2.79)** ^a^
Healthy-looking person can have HIV			
Yes	61 (62.9)	161 (83.0)	1
No	36 (37.1)	33 (17.0)	**2.88 (1.65,5.02)** ^a^
Person can get HIV from mosquito bites			
Yes	32 (33)	48 (24.7)	1.5 (0.88,2.56)
No	65 (67)	146 (75.3)	1
Person can get HIV by sharing meal with infected patients			
Yes	12 (12.4)	17 (8.8)	1.47 (0.67,3.22)
No	85 (87.6)	177 (91.2)	1
Knowledge status on HIV			
Not knowledgeable	72 (74.2)	121 (62.4)	**1.74 (1.02,2.98)** ^a^
Knowledgeable^b^	25 (25.8)	73 (37.6)	1

^a^Variable showing significant association during bivariate analysis

^b^Knowledgeable on HIV/AIDS - individuals who correctly responded for all of the questions related to HIV prevention and common misconceptions

#### Knowledge and attitude towards PITC

Among the study participants, 44.3 % of cases and 12.4 % of controls were ever not heard of PITC. However, majority of the study participants knew that patients have the right to decline HIV test (95.9 % for cases and 96.9 % for controls), supported provision of PITC service to all OPD patients (58.8 % for cases and 93.8 % for controls) and perceived the benefits of PITC (59.8 % for cases and 95.4 % for controls) (Table 3).

**Table 3:** Knowledge and attitude towards PITC service by cases and controls who attended OPD of selected health facilities in Wolaita zone, Southern Ethiopia, February 2012

Variables	Case (*n* = 97) #(%)	Control (*n* = 194) #(%)	Crude OR (95 % CI)
Ever heard of PITC			
Yes	54 (55.7)	170 (87.6)	1
No	43 (44.3)	24 (12.4)	**5.64 (3.14,10.13)** ^a^
Know PITC is given to all OPD patients			
Yes	57 (58.8)	146 (75.3)	1
No	40 (41.2)	48 (24.7)	**2.14 (1.27,3.59)** ^a^
Patient has right to refuse PITC			
Yes	93 (95.9)	188 (96.9)	1
No	4 (4.1)	6 (3.1)	1.35 (0.37,4.89)
Patients are benefited from PITC service			
Yes	89 (91.8)	187 (96.4)	1
No	8 (8.2)	7 (3.6)	2.4 (0.84,6.83)
Support provision of PITC service to all OPD patients			
Yes	57 (58.8)	182 (93.8)	1
No	40 (41.2)	12 (6.2)	**10.64 (5.23,21.66)** ^a^
Perceive the benefits of PITC			
Yes	58 (59.8)	185 (95.4)	1
No	39 (40.2)	9 (4.6)	**13.82 (6.32,30.23)** ^a^
PITC can affect health service utilization by patients			
No	81 (83.5)	181 (93.3)	1
Yes	16 (16.5)	13 (6.7)	**2.75 (1.26,5.98)** ^a^

^a^Variable showing significant association during bivariate analysis

#### Practice of HIV test

Among the study participants, 55(56.7 %) of cases and 132(68 %) of controls had ever been tested for HIV prior to our study. From those who ever been tested before, 40 % of cases (*n* = 55) and 51.5 % of controls (*n* = 132) were tested by PITC approach.

#### Perceived risk to HIV infection

Thirty-four percent of cases and 6.2 % of controls perceived themselve being at risk for HIV infection. Qualitative findings also revealed that those patients who perceived themselve being at higher risk to acquire HIV infection due to multiple sexual partner and/or unprotected sexual practice refused HIV test than individuals with no risk perception.

#### Perceived benefits of HIV testing

About 59.8 % of cases and 95.4 % of controls perceived the benefits of provider-initiated HIV testing and counseling. During qualitative study, a 28 years old male OPD clinician from Otona hospital said that *those who have information on available services in our hospital (curative, preventive and supportive services), if they were found to be positive, did not refuse providers’ recommendation as compared to those who have no information on it.*

#### Stigma towards PLWHAs

Our study showed that about 96.9 % of cases and 78.9 % of controls had stigmatizing idea towards PLWHAs. Qualitative study also strengthened that stigma remains the most important barrier among patients to accept providers’ recommendation for HIV test.

#### Pretest information

Concerning pretest information, study participants who did not get minimum recommended pretest information as per the PITC guidelines from their providers were 91.8 % for cases and 78.4 % for controls. The main reasons, as mentioned in qualitative study, were patient load and staff shortage. “*…..I did not face any patients who refused HIV test when pretest information were properly given but due to patient load and staff shortage, sometimes, we were obliged to skip some of pretest information; even some of the patients were not recommended at all, which might result in PITC refusal”. (32 years old, Male Physician, Ottona Hospital).*

### Multivariate analysis

In multivariate analysis, HIV related stigma, perceived risk for HIV infection, perceived benefits of PITC, pretest information related to PITC, ever heard of PITC service and residence have shown statistically significant association with outcome variable in the study area (Table 4).

**Table 4:** Independent predictors of PITC refusal by cases and controls who attended OPD of selected health facilities in Wolaita zone, Southern Ethiopia, February 2012

Variables	Case (*n* = 97) # (%)	Control (*n* = 194) # (%)	Crude OR (95 % CI)	Adjusted OR (95 % CI)
Stigma towards PLWHAs				
No	41 (42.3 %)	165 (85.1 %)	1	1
Yes	56 (57.7 %)	29 (14.9 %)	**8.40 (2.53,27.88)** ^a^	**6.09 (1.70,21.76)** ^a^
Perceived risk for HIV				
No	64 (66)	182 (93.8)	1	1
Yes	33 (34)	12 (6.2)	**7.82 (3.81,16.06)** ^a^	**5.23 (2.22,12.32)** ^a^
Perceived benefits of PITC				
Yes	58 (59.8)	185 (95.4)	1	1
No	39 (40.2)	9 (4.6)	**13.82 (6.32,30.23)** ^a^	**4.64 (1.79,12.01)** ^a^
Got minimum recommended pretest information				
Yes	8 (8.2)	37 (19.1)	1	1
No	89 (91.8)	157 (80.9)	**3.07 (1.38,6.84)** ^a^	**2.98 (1.06,8.35)** ^a^
Ever heard of PITC				
Yes	54 (55.7)	170 (87.6)	1	1
No	43 (44.3)	24 (12.4)	**5.64 (3.14,10.13)** ^a^	**2.41 (1.14,5.09)** ^a^
Residence				
Rural	45 (46.4)	108 (55.7)	1	1
Urban	52 (53.6)	86 (44.3)	1.45 (0.89, 2.37)	**2.40 (1.26,4.57)** ^a^

^a^Statistically significant at P value less than 0.05

## Discussion

Provider-initiated HIV testing and counseling (PITC) is important strategy designed to scale up HIV detection and play significant role to prevent and control its transmission. Though its implementation started since 2005, significant number of individuals still did not accept PITC recommendation in our country [22–24]. Thus, this study attempted to assess determinants of PITC refusal by OPD patients.

Despite many interventions aimed to reduce HIV related stigma, still there is deep-rooted stigmatizing attitude towards PLWHAs by community which is a great challenge to prevent and control HIV transmission. Our study showed that study participants who had stigmatizing attitude towards PLWHAs were more likely to refuse PITC than individuals who had no stigmatizing attitude towards PLWHAs [AOR = 6.09, (95 % CI: 1.70, 21.76)]. This finding was consistent with many other studies conducted in our country [21, 25] and Botswana [26] in which HIV related stigma was associated with decreased odds of having been tested for HIV. Having large proportion of people with stigmatizing idea is a great challenge to attain “zero new HIV infection” slogans since it reduces an individual’s willingness to be tested for HIV, to disclose their HIV status, and to access them for health care.

Individuals who had perceived risk to acquire HIV infection were more likely to refuse PITC than those who had no perceived risk for HIV infection [AOR = 5.23, (95 % CI: 2.22, 12.32)]. This finding was inconsistent with different studies conducted in our country on ANC attendee and TB patients such that those patients with less/no perceived risk to acquire the virus were more likely to refuse PITC than those with high perceived risk [27–29] while others showed that there was no association between risk perception and HIV test [18, 30, 31]. However, the finding was in line with the study conducted in East Gojam on TB patients such that patients with high perceived risk were more likely to refuse PITC than those with low/no perceived risk to acquire HIV infection [21]. This might be due to fear of stigma or wrong perception about the disease. It could be a great challange to control HIV transmission if those who thought them at higher risk to acquire the disease refused HIV test.

Study participants who did not perceive the benefits of HIV test for them were more likely to refuse PITC than their counterparts [AOR = 4.64, (95 % CI = 1.79, 12.01)]. This finding was in line with case control study conducted on TB patients and antenatal clinic attendees at Dil Chora Hospital [21, 31]. However, it was inconsistent with the descriptive cross sectional study conducted in Addis Ababa at STI clinic [32] and comparative cross sectional study conducted in Adama at TB patients [33]. The difference might be due difference in study participants and study design.

Clients who were not given minimum recommended pretest information as per the national PITC guidelines by their providers were more likely to refuse PITC than those who were given it [AOR = 2.98, (95 % CI: 1.06, 8.35)]. This might be due to the fact that those who did not get adequate pretest information could not understood the benefits of HIV test and thus refused PITC. As per the national PITC guidelines providers should give minimum recommended pretest information to all OPD clients seeking medical care; however they did not practice it consistently especially during high patient load. Thus offering HIV testing service to all individuals seeking health care had better been given due attention since it is key strategy to increase HIV test rate, early detection of the disease and enrollment of patients to care, treatment and support.

Those who ever not heard of PITC before contact with OPD clinicians were more likely to refuse PITC [AOR = 2.41, (95 % CI: 1.14, 5.09)]. This finding was in line with case control study conducted in East Gojam on TB patients [21]. This could be due to the fact that those who ever not heard of PITC need more time to think of HIV test and/or discuss with whom they want before accepting PITC service. A 26 years old OPD clinician from Gununo health center said that “…*those who ever not heard of PITC service did not usually show readiness for HIV test and they request extra time to discuss it with whom they want to discuss than those who ever heard of PITC”*. Thus, prior awareness creation on issues related to HIV testing should be considered before recommending PITC to clients at outpatient department in the study area.

Study participants who reside in urban areas were about twice more likely to refuse PITC than that of rural residence [AOR = 2.40, (95 % CI = 1.26, 4.57)]. This finding was inconsistent with population based study conducted in Botswana [26, 34] and facility based study conducted in our country [25, 27, 30]. This might be due to high patient flow and subsequently work load by providers in urban health facility than rural area so that urban patients did not get the minimum recommended pretest information as per the PITC guidelines to understand the benefits of HIV test.

### Strength and limitation of the study

This study addressed factors related to health care providers in addition to patients’ related factors, which will help to broaden our target intervention. However, social desirability bias related to the sensitivity of topic could affect the response of the participants.

## Conclusion

PITC service was not delivered to all OPD clients all the time in the study area especially at hospitals as per the national PITC guidelines. Lack of knowledge on HIV/AIDS, stigmatizing attitude towards people living with HIV/AIDS, low perceived benefits on provider-initiated HIV testing and counseling, perceived risk for HIV infection and residing in urban areas were major barriers for provider-initiated HIV testing and counseling acceptance in our study. Therefore, concerned bodies should strengthen IEC/BCC on importance and benefits of HIV testing, especially to urban dwellers and should design and implement different strategies to washout deep-rooted stigmatizing attitude towards PLWHAs to reduce PITC refusal by OPD patients.
